# Electrical switching between exciton dissociation to exciton funneling in MoSe_2_/WS_2_ heterostructure

**DOI:** 10.1038/s41467-020-16419-x

**Published:** 2020-05-26

**Authors:** Yuze Meng, Tianmeng Wang, Chenhao Jin, Zhipeng Li, Shengnan Miao, Zhen Lian, Takashi Taniguchi, Kenji Watanabe, Fengqi Song, Su-Fei Shi

**Affiliations:** 1 Department of Chemical and Biological Engineering, Rensselaer Polytechnic Institute, Troy, NY 12180 USA; 2 National Laboratory of Solid State Microstructures, Collaborative Innovation Center of Advanced Microstructures, and School of Physics, Nanjing University, 210093 Nanjing, P. R. China; 3 Kavli Institute at Cornell for Nanoscale Science, Cornell University, Ithaca, NY 14853 USA; 4 National Institute for Materials Science, 1-1 Namiki, Tsukuba, 305-0044 Japan; 5 Department of Electrical Computer & Systems Engineering, Rensselaer Polytechnic Institute, Troy, NY 12180 USA

**Keywords:** Two-dimensional materials, Two-dimensional materials

## Abstract

The heterostructure of monolayer transition metal dichalcogenides (TMDCs) provides a unique platform to manipulate exciton dynamics. The ultrafast carrier transfer across the van der Waals interface of the TMDC hetero-bilayer can efficiently separate electrons and holes in the intralayer excitons with a type II alignment, but it will funnel excitons into one layer with a type I alignment. In this work, we demonstrate the reversible switch from exciton dissociation to exciton funneling in a MoSe_2_/WS_2_ heterostructure, which manifests itself as the photoluminescence (PL) quenching to PL enhancement transition. This transition was realized through effectively controlling the quantum capacitance of both MoSe_2_ and WS_2_ layers with gating. PL excitation spectroscopy study unveils that PL enhancement arises from the blockage of the optically excited electron transfer from MoSe_2_ to WS_2_. Our work demonstrates electrical control of photoexcited carrier transfer across the van der Waals interface, the understanding of which promises applications in quantum optoelectronics.

## Abstract (web summary)

The ultrafast carrier dynamics across the van der Waals interface of transition metal dichalcogenide heterostructures govern the formation and funnelling of excitons. Here, the authors demonstrate a reversible switch from exciton dissociation to exciton funnelling in a MoSe_2_/WS_2_ heterostructure, which manifests itself as a photoluminescence quenching-to-enhancement transition.

## Introduction

Two-dimensional (2D) semiconductors are promising candidates for light-harvesting and optoelectronic applications^1–5^ due to their strong light–matter interaction from excitonic responses^6–13^. Their atomically thin nature further enables engineering exciton dynamics and energy relaxation pathways through ultrafast carrier transfer across 2D van der Waals (vdW) interfaces^14–21^. In particular, a vdW heterostructure can, respectively, dissociate electrons and holes into separate layers or funnel excitons to one layer with a type II or type I band alignment^15,21–28^. It is highly desirable to achieve both functions in a single device in an electrically reconfigurable way. However, to the best of our knowledge, this has not been demonstrated yet. Here we demonstrate reversible electrical switching between exciton dissociation and funneling in a MoSe_2_/WS_2_ heterostructure device. We show that the electron transfer from MoSe_2_ to WS_2_ can be blocked by efficient gating of the LaF_3_ substrate, leading to a transition between photoluminescence (PL) quenching to PL enhancement for the MoSe_2_ A exciton emission. The ability to electrically control interlayer charge transfer pathways ushers in application concepts, such as light switch and energy steering.

## Results

### Charge transfer in the MoSe_2_/WS_2_ heterostructure

**Fig. 1:**
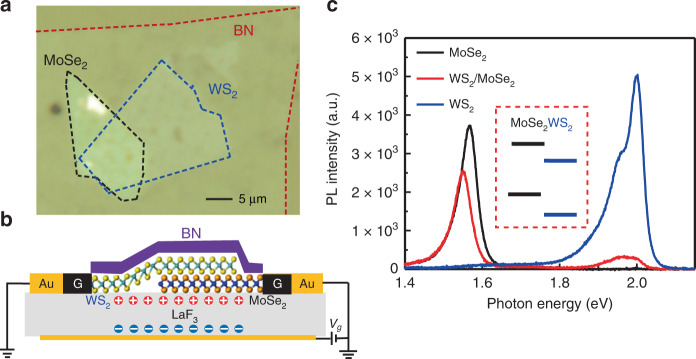
Monolayer MoSe_2_/WS_2_ heterostructure device. **a** Optical microscopic image of the monolayer MoSe_2_/WS_2_ heterostructure, capped with a few-layer h-BN layer. Scale bar: 5 µm. **b** Schematic of the MoSe_2_/WS_2_ heterostructure device, contacted by few-layer graphene electrodes and gated by the ionic substrate LaF_3_. **c** Typical room temperature PL spectra from regions of the monolayer MoSe_2_ (black), monolayer WS_2_ (blue), and MoSe_2_/WS_2_ heterojunction (red), with no gate voltage applied. Inset: schematic representation of the type II band alignment of the MoSe_2_/WS_2_ heterostructure.

We construct the MoSe_2_/WS_2_ heterostructure on the LaF_3_ substrate through a layer-by-layer dry transfer technique^29^, and the heterostructure is also capped by a thin layer of hexagonal boron nitride (BN) on the top. A typical MoSe_2_/WS_2_ heterostructure on the LaF_3_ substrate is shown in Fig. 1a. The overlapped region of the monolayer MoSe_2_ and WS_2_ forms the MoSe_2_/WS_2_ heterojunction. We use few-layer flakes of graphene to contact both the monolayer MoSe_2_ and WS_2_, and a schematic of the device is shown in Fig. 1b. The heterostructure can be gated through the LaF_3_ substrate as the back gate, which provides efficient control of doping through the double layer^30^, as schematically shown in Fig. 1b. Typical PL spectra for different regions of the device are shown in Fig. 1c, with the continuous wave (CW) laser excitation centered at 2.331 eV and a power of 100 µW. Without applying any gate voltage, the PL from the MoSe_2_/WS_2_ heterojunction (red line in Fig. 1c) exhibits quenching of both the PL at the WS_2_ A exciton resonance (~1.979 eV) and MoSe_2_ A exciton resonance (~1.548 eV), compared with that of the monolayer WS_2_ (blue line in Fig. 1c) and the monolayer MoSe_2_ (black line in Fig. 1c), respectively (see Supplementary Note 4). This simultaneous quenching of PL at both MoSe_2_ and WS_2_ A excitons was observed in all the heterostructures we constructed, including three MoSe_2_/WS_2_ heterostructures on SiO_2_/Si substrate and seven heterostructures on LaF_3_ in the absence of the gate voltage (see Supplementary Notes 1 and 7). It thus suggests a type II alignment for the as-prepared MoSe_2_/WS_2_ heterostructures, and the PL quenching is a result of the optically excited electron transferred to the MoSe_2_ layer and hole transferred to the WS_2_, according to the band alignment^31,32^ shown schematically in the inset of Fig. 1c. It is interesting to note that the quenching of MoSe_2_ A exciton PL is significantly less than that of the WS_2_ A exciton in the heterojunction region. While the integrated PL of the WS_2_ A exciton in the heterojunction is quenched by more than one order of magnitude smaller, the integrated PL intensity of MoSe_2_ A exciton in the heterojunction is only slightly quenched, being ~70% of that from the monolayer MoSe_2_ (Fig. 1c). The significantly less quenching of MoSe_2_ A exciton PL can be understood from the relative band alignment shown in the inset of Fig. 1c. In the type II alignment configuration, the conduction band minimum (CBM) of the WS_2_ is only slightly lower than that of the MoSe_2_ according to the theoretical calculations^1,32^. The thermal equilibrium of the two CBMs at room temperature therefore allows a certain population of electrons in the CBM of the MoSe_2_ even though the CBM of the WS_2_ is the lower energy state for electrons in the heterojunction region. The small energy difference between the two CBMs offers the opportunity for us to apply an efficient electrostatic gating to manipulate the optically excited carrier transfer across the MoSe_2_/WS_2_ interface. We achieve that by using the LaF_3_ as the ionic back gate, which has been proven to efficiently gate 2D materials though a double layer^30^ (schematically shown in Fig. 1b).

### Gate-dependent PL enhancement in MoSe_2_/WS2

**Fig. 2:**
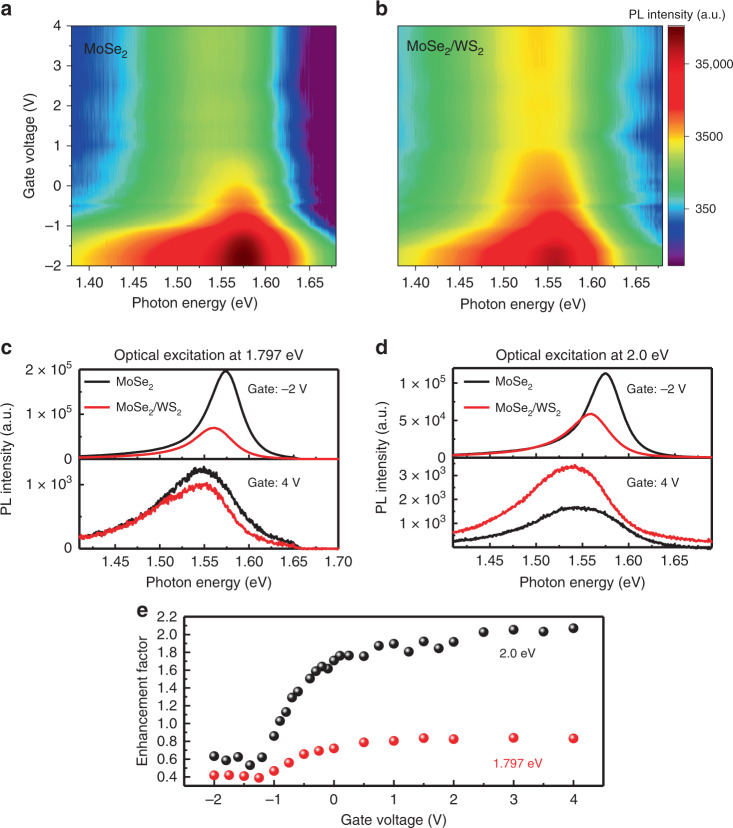
Gate voltage-tunable transition from PL quenching to PL enhancement in the MoSe_2_/WS_2_ heterojunction. **a** The color plot of the PL spectra for monolayer MoSe_2_ and **b** the color plot of the PL spectra for MoSe_2_/WS_2_ heterostructure region as a function of the gate voltage, under the continuous wave (CW) photoexcitation centered at 2.0 eV and with the excitation power of 100 µW. The color represents the integrated PL intensity at MoSe_2_ A exciton resonance. All spectra were taken at room temperature. **c**, **d** are PL spectra of the monolayer MoSe_2_ (black) and MoSe_2_/WS_2_ heterojunction (red) under the CW photoexcitation centered at 1.797 and 2.0 eV, respectively. The excitation power for both **c** and **d** is 100 µW. **e** The experimentally extracted PL enhancement factor as a function of the gate voltage for the photoexcitation centered at 2.0 eV (black dots) and 1.797 eV (red dots).

To reveal the effect of gate-controlled carrier transfer across the heterojunction, we then investigate the PL spectra around the MoSe_2_ A exciton resonance as a function of the gate voltage for both monolayer MoSe_2_ (Fig. 2a) and MoSe_2_/WS_2_ heterojunction (Fig. 2b) (see Supplementary Note 5). The CW laser excitation centered at 2.0 eV (620 nm) with a power of 100 µW was used to obtain the PL spectra shown in Fig. 2a, b. This excitation photon energy is large enough to excite A excitons in both monolayer MoSe_2_ (1.548 eV) and WS_2_ (1.979 eV). The PL intensity from the WS_2_ A exciton is drastically quenched in the heterojunction, and we focus on the PL intensity of the MoSe_2_ A exciton for both the monolayer (Fig. 2a) and heterojunction region (Fig. 2b). We can see from the color plots (Fig. 2a, b) that, although the MoSe_2_ A exciton PL intensity is weaker in MoSe_2_/WS_2_ heterojunction (Fig. 2b) than in the monolayer MoSe_2_ (Fig. 2a) for the gate voltage from ~−2 to −1 V, the PL is stronger in the heterojunction than in the monolayer MoSe_2_ at the gate voltage >0 V. This relative PL ratio from quenching to enhancement transition is clearly illustrated in the PL spectra in Fig. 2d, which combine the line cuts of Fig. 2a, b at the gate voltage −2 and 4 V. To better understand the PL behavior change, we define the PL enhancement factor (EF) as ${\rm{EF}} = I_{{\rm{Heter}}}/I_{{\rm{MoSe}}_2}$, where *I*_Heter_ ($I_{{\rm{MoSe}}_2}$) is the integrated MoSe_2_ A exciton PL intensity in the MoSe_2_/WS_2_ heterojunction (monolayer MoSe_2_). EF as a function of the gate voltage for the photoexcitation centered at 2.0 eV is shown in Fig. 2e (black dots), which shows that EF is almost a constant between the gate voltage of −2 to −1 V (EF ~ 0.6) but quickly rises to ~1.8 at the gate voltage 0 V, and it remains largely a constant as the gate voltage is further increased.

It is interesting to note that this observation is sensitive to the excitation photon energy, and the results are distinctively different for the CW photoexcitation of the same power (100 µW) but centered at 1.797 eV (690 nm), which is below the A exciton resonance energy of WS_2_ but above that of MoSe_2_. At the gate voltage of −4 V, we observe PL quenching at MoSe_2_ A exciton resonance in the heterojunction (Fig. 2d), similar to the scenario with the photoexcitation at 2 eV (Fig. 2c). However, as we increase the gate voltage to 4 V, we do not observe the PL enhancement of the MoSe_2_ A exciton in the heterojunction, even though the PL intensity is quite close to that of the monolayer MoSe_2_ (Fig. 2d). A detailed gate-dependence study of the photoexcitation centered at 1.797 eV also results in quantitative EF as shown in Fig. 2e (red dots), which shows a similar step function behavior as the case of photoexcitation centered at 2.0 eV, but the maximum value of EF is smaller and never exceeds 1.

### PL excitation (PLE) spectroscopy of EF in MoSe_2_/WS_2_

**Fig. 3:**
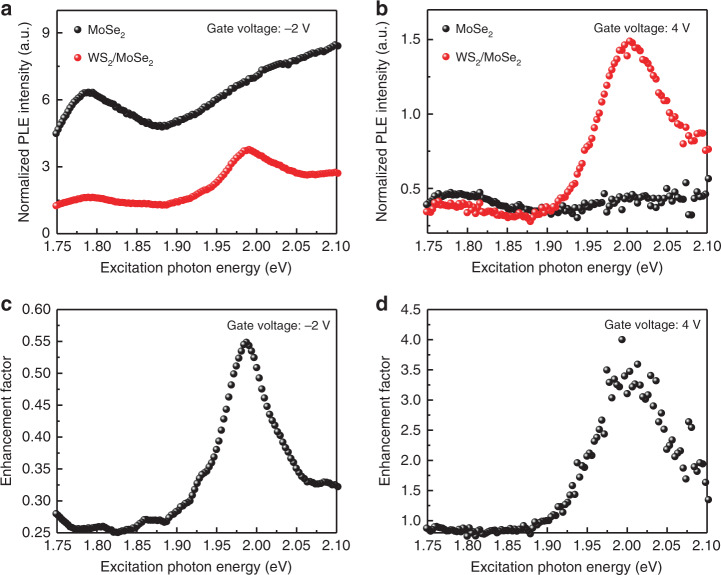
PLE spectra of the monolayer MoSe_2_ and MoSe_2_/WS_2_ heterostructure for different gate voltages. **a**, **b** are integrated PL intensity at MoSe_2_ A exciton resonance as a function of the excitation photon energy for monolayer MoSe_2_ (black) and MoSe_2_/WS_2_ heterojunction (red) regions at the gate voltage of −2 V (**a**) and the gate voltage of 4 V (**b**), respectively. **c**, **d** are PL enhanced factor for the gate voltage of −2 V (**c**) and 4 V (**d**), respectively.

Since the observed PL EF at MoSe_2_ A exciton resonance is sensitive to the excitation photon energy, we then perform a detailed PLE spectroscopic study. The integrated PL intensity at the MoSe_2_ A exciton resonance for monolayer MoSe_2_ (black) and MoSe_2_/WS_2_ heterojunction (red) are plotted as a function of the excitation photon energy in Fig. 3a, b for the gate voltage of −2 and 4 V, respectively. Figure 3c shows the EF for the gate voltage of −2 V, and when both MoSe_2_ and WS_2_ are intrinsic, the MoSe_2_ A exciton PL intensity is always quenched for different excitation photon energies, with the peak value of 0.55 at the excitation photon energy of ~2.0 eV. However, at the gate voltage of 4 V, the EF is largely flat and slightly <1 (~0.8) when the excitation photon energy is <1.9 eV. When the excitation photon energy >1.9 eV, the PL enhancement effect starts to occur with the EF >1. The EF reaches the maximum value when the excitation photon energy is about 2.0 eV. The excitation photon energy for the peaked EF value in Fig. 3c, d coincides with the A exciton resonance of monolayer WS_2_, which suggests that photoexcited carrier transfer from WS_2_ to MoSe_2_ plays the central role in the PL enhancement in the MoSe_2_/WS_2_ heterojunction, which, as a result, explains the lack of the PL enhancement with the off-resonance excitation at 1.797 eV (Fig. 2d).

It is worth noting that, even with the CW photoexcitation centered at 2.0 eV, we have not observed that the PL EF exceeds 1 for three MoSe_2_/WS_2_ heterojunction devices fabricated on SiO_2_/Si substrate (300-nm-thick thermal oxide), with the silicon back gate voltage as high as 80 V (see Supplementary Note 1). This observation suggests that the efficient gating from LaF_3_ is essential for realizing the PL enhancement in the heterojunction. Previous work has shown that the LaF_3_ back gate should be at least >100 times more efficient than the silicon back gate with 300 nm thermal oxide^30^.

## Discussion

**Fig. 4:**
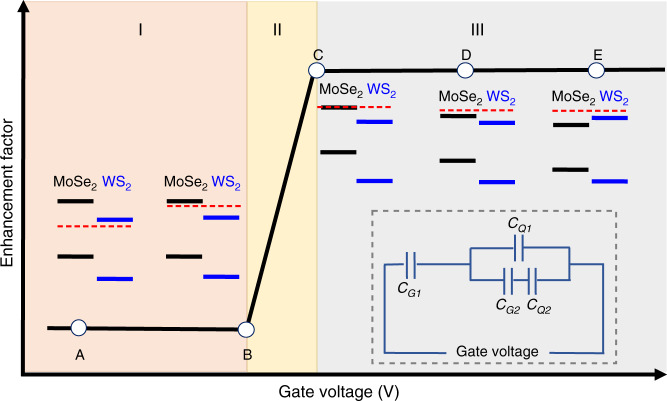
Theoretical understanding of the PL quenching to PL enhancement transition. The enhancement factor as a function of the gate voltage clearly exhibits three distinct regions. Schematics of band alignment of the MoSe_2_/WS_2_ heterojunction, along with the Fermi energy level (dashed line), are labeled for different points to explain the different PL enhancement factor behaviors. Inset: schematic of the effective capacitance circuit of the MoSe_2_/WS_2_ heterojunction with the device configuration shown in Fig. 1b.

The experimental observation can be understood theoretically by considering the gate-dependent carrier distribution in the heterostructure. Taking into account the quantum capacitance of the monolayer MoSe_2_ and WS_2_, for the device configuration shown in Fig. 1b, the effective capacitance model can be schematically shown as the inset of Fig. 4 (MoSe_2_ being the bottom layer, and detailed derivation in Supplementary Note 3). Here *C*_*Q*1_(*C*_*Q*2_) are the quantum capacitance of monolayer MoSe_2_ (WS_2_), *C*_*G*1_ is the geometry capacitance between MoSe_2_ and the LaF_3_ back gate, and *C*_*G*2_ is the geometry capacitance between MoSe_2_ and WS_2_. For qualitative understanding, we consider zero-temperature case here (see Supplementary Note 2 for the discussion of the finite temperature case, which does not qualitatively change the picture). Owing to the large energy difference between the VBMs of MoSe_2_ and WS_2_, the hole transfer from WS_2_ to MoSe_2_ (when WS_2_ is optically excited) is always ~100%. As a result, we focus on the gate dependence of the electron transfer from MoSe_2_ to WS_2_. As shown in Fig. 4, when the gate voltage is at point A (e.g., −2 V for the device 2 shown in Fig. 2), both the MoSe_2_ and WS_2_ layers are intrinsic and with the quantum capacitance of zero. As a result, the gate voltage is dropped only on the quantum capacitance and the band alignment is determined by the work function of each layer. The type II alignment (shown at point A in Fig. 4) determines that the optically excited electron in MoSe_2_ will transfer to WS_2_, reducing the electron density in the MoSe_2_ layer in the heterojunction, compared to the case of the bare monolayer MoSe_2_. In addition, with (on-resonance excitation) and without (off-resonance excitation) the hole transfer from WS_2_ to MoSe_2_, the electron density in the MoSe_2_ layer in the heterojunction is always less than the hole density. As a result, the electron is the minor carrier that determines the available MoSe_2_ A exciton density. The reduced electron density thus leads to the quenching of MoSe_2_ A exciton PL in the heterojunction.

When the WS_2_ layer starts to get electron-doped (point B), the number of optically excited electrons transferred from the MoSe_2_ to WS_2_ in the heterojunction region will be modulated by the gate voltage. For simplicity, we can use the off-resonance excitation as an example. The charge transfer from MoSe_2_ to WS_2_, Δ*Q*, can be obtained from the following equation according to the effective capacitance model^33^ (inset of Fig. 4):

1 $$\frac{{Q - {\mathrm{\Delta }}Q}}{{C_{Q1}}} = \frac{{{\mathrm{\Delta }}Q}}{{C_{Q2}}} + \frac{{{\mathrm{\Delta }}Q}}{{C_{G2}}},$$

where *Q* is the total charge of optically excited electrons in the MoSe_2_ layer of the MoSe_2_/WS_2_ heterojunction. Reorganization of Eq. (1) results in the expression of Δ*Q* as:

2 $$\frac{{{\mathrm{\Delta }}Q}}{Q} = \frac{1}{{1 + \frac{{C_{Q1}}}{{C_{Q2}}} + \frac{{C_{Q1}}}{{C_{G2}}}}}.$$

For gate voltage smaller than that of point B, *C*_*Q*1_ = 0 and hence Δ*Q* = *Q*, which indicates that ~100% of the optically excited electron in MoSe_2_ layer of the heterojunction region is transferred to WS_2_. As a result, PL quenching of the MoSe_2_ layer in the heterojunction is similar to that of A point (similar EF). As we move forward from point B, however, electron transfer will be less efficient due to the finite *C*_*Q*1_ (i.e., finite density of states (DOS) at Fermi level in MoSe_2_) and the PL quenching will be less significant. As the gate voltage is increased to point C, the doping further increases and the Fermi level is aligned with the conduction band of MoSe_2_. Assuming a similar effective electron mass *m* in WS_2_ and MoSe_2_, we have *C*_*Q*1_ = *C*_*Q*2_ = *C*_*Q*_, where $C_Q = \frac{m}{{\pi \hbar ^2}}$ is the DOS in 2D. Since *C*_*Q*_ ≫ *C*_*G*2_ (see Supplementary Note 2), from Eq. (2), we found that Δ*Q* ~ 0 and optically excited electron transfer from MoSe_2_ to WS_2_ is blocked. The EF of MoSe_2_ A exciton PL will therefore again be largely a constant, with the value of 1 (Fig. 4) for the off-resonance excitation in the ideal scenario.

The electron transfer in the on-resonance scenario can be understood in a similar fashion (see Supplementary Note 2), with similar PL quenching (EF < 1) from point A to B. However, when MoSe_2_ is sufficiently doped (point C), optically induced holes in the MoSe_2_ layer become the minor carrier that determines the MoSe_2_ A exciton density. For the on-resonance excitation, the WS_2_ layer is also excited and we have nearly 100% of the optically excited holes transfer from WS_2_ to MoSe_2_. Therefore, the A exciton density in the MoSe_2_ layer in the heterojunction is enhanced, giving rise to the PL enhancement with a largely constant EF > 1. We thus conclude that, for both the off-resonance and on-resonance excitation, the qualitative gate dependence of EF will be of the form shown in Fig. 4. Particularly, EF will show an abrupt increase around specific gate voltage (region II) and remain largely constant on either side. On the low voltage side (region I), EF should be <1; and on the high gate voltage side (region III), EF = 1 for the off-resonance excitation and EF > 1 for the on-resonance excitation.

The theoretical prediction in Fig. 4 is in excellent agreement with our experimental observation in Fig. 2e. The experimentally observed EF as a function of the gate voltage can be clearly divided into three regions, similar to a step function for both the on-resonance and off-resonance excitation as predicted by Fig. 4. The EF for the on-resonance excitation (photoexcitation at 2.0 eV) in region III shows an EF ~ 2.0, while the EF for the off-resonance excitation (photoexcitation at 1.797 eV) in the region III is about 0.8. The EF of <1 for the off-resonance case is probably due to decreased quantum efficiency in the heterojunction from the different dielectric environment.

The consideration of the finite temperature effects is included in Supplementary Note 2, and it gives qualitatively similar results as in Fig. 4. Interestingly, we found that, for large enough gate voltage, the charge accumulated on the *C*_*G*2_ will give rise to a large energy shift between the MoSe_2_ and WS_2_, which switches the type II alignment to a type I alignment configuration, as shown schematically by the inset at point E in Fig. 4. The efficient ionic gating thus not only allows the control of optically excited carrier transfer across the atomically sharp interface but also leads to the possibility of modifying the alignment type reversibly. The associated fundamental understanding will enable quantum optoelectronics based on transition metal dichalcogenide (TMDC) vdW heterostructures.

## Method

### Device fabrication

The MoSe_2_/WS_2_ heterostructure devices were fabricated through a layer-by-layer dry transfer technique^29^. More specifically, each of the monolayer TMDC was sequentially transferred to the LaF_3_ substrate, and a final BN flake was used to cap the heterostructure. Two pieces of few-layer graphene were used as the electrodes to contact the monolayer MoSe_2_ and WS_2_ layer separately, and both were grounded during the measurements, as schematically shown in Fig. 1b. The final devices were annealed in vacuum at 100 °C for 3 h.

### Optical measurements

All the optical measurements in this work were performed at room temperature. The micro-PL measurements were performed with a home-built confocal microscope, in which the excitation lasers were focused to a spot size of ~2 µm. The PLE spectra were taken with a supercontinuum white laser (Fianium), and the filtered light (with bandwidth ~4 nm) was used as the excitation source.

## Supplementary information

Supplementary Information

Peer Review File

## Data Availability

The data that support the findings of this study are available from the authors on reasonable request, see “Author contributions” for specific data sets.
