# Supplementary material for: Electrical switching between exciton dissociation to exciton funneling in MoSe2/WS2 heterostructure
Source: Nat Commun. 2020 May 26;11:2640. doi: 10.1038/s41467-020-16419-x (PMC7250925; doi:10.1038/s41467-020-16419-x)
Supplement: Supplementary file 1 — Supplementary Information [file 41467_2020_16419_MOESM1_ESM.pdf]

Supplementary Information

Electrical Switching between Exciton Dissociation to Exciton Funneling in $MoSe_2/WS_2$ Heterostructure

Meng et al.

Supplementary Note 1. $MoSe_2/WS_2$ heterostructure devices on SiO_2/Si substrate.

We have fabricated $MoSe_2/WS_2$ heterostructure devices on SiO_2/Si substrate with the same dry transfer method[1] as control, and the device structure is schematically shown in Supplementary Fig. 1b. The optical microscope image of one typical device is shown in Supplementary Fig. 1, which clearly shows three distinctive regions: monolayer $MoSe_2$, monolayer WS_2, and $MoSe_2/WS_2$ heterojunction.

Supplementary Fig. 1c shows PL spectra of monolayer $MoSe_2$ (red), monolayer WS_2 (blue) and WS_2/ $MoSe_2$ heterojunction(black) at room temperature without applying the gate voltage. A continuous-wave (CW) laser centered at 532 nm with the power of 100 μW was used as the excitation. It is evident that both the $MoSe_2$ A exciton and WS_2 A exciton PL signals are quenched in the heterojunction, suggesting a type II alignment in the $MoSe_2/WS_2$ heterojunction.

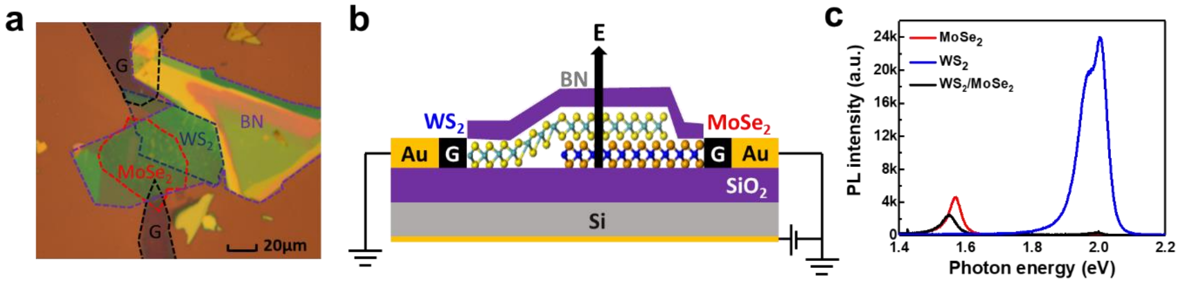

Supplementary Figure 1. (a) Optical microscope image of one WS_2/ $MoSe_2$ heterostructure covered by BN on SiO_2/Si substrate. scale bar: 20 μm. (b) Schematic of the WS_2/ $MoSe_2$ heterostructure device. (c) PL spectrum of monolayer $MoSe_2$ (red), monolayer WS_2 (blue) and WS_2/ $MoSe_2$ heterojunction(black) with no gate voltage applied.

For the heterostructure device on SiO_2/Si substrate, the gate dependence of the PL spectra is shown in Supplementary Fig. 2a and 2b, with the photoexcitation centered at 2.33 eV and the excitation power of 100 μW. For the back-gate voltage from -80 V to 80 V, no PL enhancement was observed for the $MoSe_2$ A exciton, different from what was observed on the devices on LaF_3 (shown in the main text). The integrated PL intensity at $MoSe_2$ A exciton resonance as a function of the back gate is shown in Supplementary Fig. 2d and the associated enhancement factor (EF) is shown in Supplementary Fig. 2e.

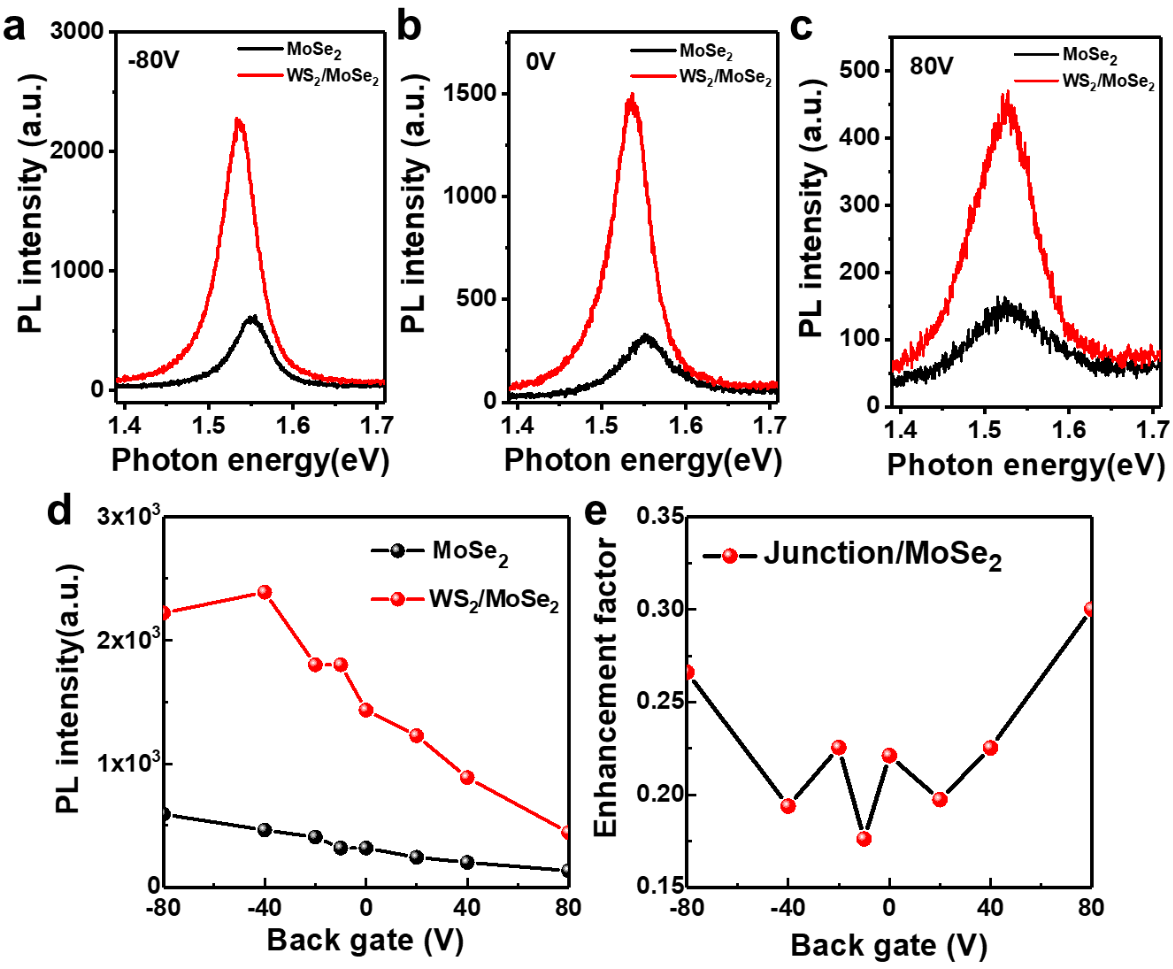

Supplementary Figure 2. (a),(b), and (c) are PL spectra of $WS_2/MoSe_2$ heterojunction and monolayer $MoSe_2$ on SiO_2/Si substrate for the back gate voltage of -80 V, 0 V and 80 V, respectively. (d) Integrated PL intensity at $MoSe_2$ A exciton resonance for monolayer the monolayer $MoSe_2$ (black) and heterojunction(red) area as a function of the back-gate voltage. (e) Calculated PL enhancement factor (EF) as a function of the back gate.

Supplementary Note 2. Theoretical calculation of the PL enhancement factor and Fermi level as a function of gate voltage for the heterostructure devices on LaF_3.

For the device configuration shown as Fig. 1b, we have established the equivalent circuit model for our heterojunction as shown in Supplementary Fig. 3, where C_{G1} is the geometrical capacitance between the back gate and $MoSe_2$, and C_{G2} is the geometrical capacitance between the $MoSe_2$ and WS_2. C_{Q1}, C_{Q2} are the quantum capacitance of $MoSe_2$ and WS_2 monolayers, respectively. The PL from $MoSe_2$ A exciton in the heterojunction is determined by the $MoSe_2$ A exciton density. When the electron and holes density are no equal, either due to initial doping or charge transfer, the $MoSe_2$ A exciton density in the heterojunction is determined by the minor carrier (electron or hole, whichever has the smaller density).

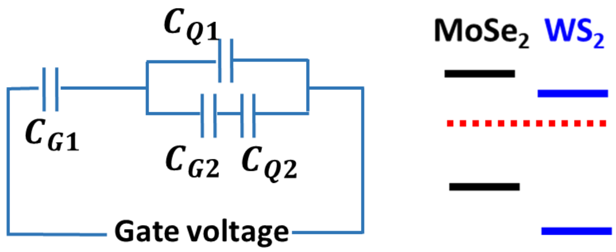

Supplementary Figure 3. (a) Effective capacitance circuit model of the heterostructure device. (b) Scheme of band alignment of the heterojunction for both layers being intrinsic.

We study three different regions, controlled by the gate voltage, for the different EF response from the heterojunction: (I) both the $MoSe_2$ and WS_2 monolayers are intrinsic; (II) the $MoSe_2$ layer is intrinsic while the WS_2 layer is n-doped, (III) both the $MoSe_2$ and WS_2 layers are n-doped.

First, for the region (I), both $MoSe_2$ and WS_2 are charge-neutral, and the Fermi level lies within their bandgap. In this case, the quantum capacitances from both layers are small (0 at 0 K). From the circuit model (Supplementary Fig. 3), all voltages will drop on the quantum capacitances, and there will be no free carriers induced by the gating. Therefore, all free carriers (i.e., both electrons and holes) in the system are generated by optical excitation. For the photoexcitation centered at 1.797 eV (690 nm), there is no optical excitation of the WS_2, and we only need to consider electron transfer from $MoSe_2$ to WS_2. As a result, the optically generated electron will have less density than the optically excited hole, and the PL intensity will be determined by the remaining electron density in the $MoSe_2$ after the carrier transfer. This electron transfer is not affected by the gate voltage until the Fermi level is raised to the conduction band of WS_2. Therefore, we would expect a largely constant EF with a value less than 1 in this region.

Secondly, in the region (II), the Fermi level is raised above the Fermi level of WS_2 to introduce electron doping in the WS_2 layer, while the $MoSe_2$ is still intrinsic. After the optical excitation, $MoSe_2$ will have more electrons than the case in the region (I) because there will be less optically excited electrons transferred to WS_2. Therefore, the EF will increase. This regime ends when the electron density in $MoSe_2$ exceeds the hole density in $MoSe_2$, controlled by the increase of the gate voltage (holes are all optically excited and do not depend much on gate voltage).

Third, for the region (III), when the electron density in $MoSe_2$ exceeds the optically excited hole density, the PL intensity will be limited by the hole density, which is almost a constant for both off-resonance and on-resonance excitation. For the on-resonance excitation, the hole transfer from WS_2 to $MoSe_2$ can be viewed as nearly 100% and not sensitive to the gate voltage, considering the large band offset between the VBM of WS_2 and $MoSe_2$ Therefore, the EF will again be a constant, which should be 1 for

the off-resonance excitation centered at 1.797 eV excitation in the ideal scenario and larger than 1 for the on-resonance excitation at 2.0 eV. There will be more holes in the $MoSe_2$ layer in the latter case, considering the hole transfer from WS_2 to $MoSe_2$. As a result, the $MoSe_2$ A exciton density will be larger in the hetero-junction compared with the $MoSe_2$ monolayer.

Next, we will quantitatively determine the proportion of the electrons transferred to WS_2, when the optical excitation generates electron and hole in the $MoSe_2$ at a given band alignment and chemical potential. The answer can be easily generalized to hole transfer or to charge transfer from WS_2 to $MoSe_2$.

Define the following "number capacitance."

$$C_{Q1} = \frac{dn_1}{d\mu_1}, C_{Q2} = \frac{dn_2}{d\mu_2}, C_{G2} = \frac{C_{g2}}{eS} \quad (1)$$

n_1, n_2 are the electron density in $MoSe_2$ and WS_2, μ_1 and μ_2 are the chemical potential counting from the conduction band edge of each material, S is the area of the device, and e is the electron charge. These "number capacitance" basically tells how much electron density is changed from the change of chemical potential or electrical potential.

The charge transfer process can be understood in the following, taking the excitation centered at 1.797 eV as an example. Prior to the optical excitation, the two layers are in equilibrium so that their chemical potential is aligned. Optically injected electrons raise the chemical potential in $MoSe_2$, breaking the equilibrium. After the electrons transfer from $MoSe_2$ to WS_2, the chemical potential for electrons becomes aligned again. Considering the electron transfer, the final equilibrium condition is described by:

$$\frac{Q-\Delta Q}{C_{Q1}} = \frac{\Delta Q}{C_{Q2}} + \frac{\Delta Q}{C_{G2}} \quad (2),$$

where Q is the optically excited electron density, ΔQ is the electron transferred from $MoSe_2$ to WS_2. The second term on the right-hand side corresponds to the fact that the charge imbalance between the two layers, arising from the charge transfer, induces an electrical potential difference (or band alignment change).

For the on-resonance excitation centered at 2.0 eV, the hole transferred from WS_2 to $MoSe_2$ needs to be considered, which we assume to be 100%. The Equation (2) needs to be modified as follows:

$$\frac{Q-\Delta Q}{C_{Q1}} = \frac{\Delta Q}{C_{Q2}} + \frac{\Delta Q+Q}{C_{G2}}.$$

However, the qualitative behavior of EF will not change. Without loss of generality, we restrict our discussion to the off-resonance excitation centered at 1.797 eV. We thus have:

$$\frac{\Delta Q}{Q} = \frac{1}{1 + \frac{C_{Q1}}{C_{Q2}} + \frac{C_{Q1}}{C_{G2}}} \quad (3)$$

In the region where the PL is dominated by the density of electrons, we have $EF \propto 1 - \frac{\Delta Q}{Q}$.

At equilibrium, we have

$$\mu_1 = \mu - \Delta E_C$$

$$\mu_2 = \mu$$

$$\Delta E_C = \Delta E_{C0} - \frac{n_2}{C_{G2}} \quad (4)$$

ΔE_C is the conduction band offset between $MoSe_2$ and WS_2, ΔE_{C0} is the initial conduction band offset ($MoSe_2$ minus WS_2), and μ is the chemical potential of the system counting from the conduction band of WS_2.

We can now be more quantitative and obtain the expression for all the capacitance at the finite temperature T.

$$n_1 = C_Q k_B T ln\left(1 + e^{\frac{\mu - \Delta E_C}{k_B T}}\right) \quad (5)$$

$$n_2 = C_Q k_B T ln\left(1 + e^{\frac{\mu}{k_B T}}\right) \quad (6)$$

$$C_{Q1} = C_Q \frac{e^{\frac{\mu - \Delta E_C}{k_B T}}}{1 + e^{\frac{\mu - \Delta E_C}{k_B T}}} \quad (7)$$

$$C_{Q2} = C_Q \frac{e^{\frac{\mu}{k_B T}}}{1 + e^{\frac{\mu}{k_B T}}} \quad (8)$$

Here $C_Q = \frac{me}{\pi \hbar^2}$ is the 2D density of states (DOS), and we assume the two layers have the same electron mass $m = 0.4\, m_0$. m_0 is the free electron mass.

The geometrical number capacitance between WS_2 and $MoSe_2$ is given by:

$$C_{G2} = \frac{\epsilon_0 \epsilon_2}{de} \quad (9)$$

where d = 1.3 nm is the distance between the $MoSe_2$ and WS_2. We use[2] $\epsilon_2 = 6.85$ as the average dielectric constant for the monolayer $MoSe_2$ and WS_2. Numerically we have

$$C_Q = 1.67 \times 10^{14} cm^2/eV$$

$$C_{G2} = 2.91 \times 10^{13} cm^2/eV$$

Considering the EF is ~0.4 for the region (I) for off-resonance excitation at 1.797 eV (Fig. 2e in the main text) and EF is ~0.8 for the region (III), we expect the quantum

efficiency for the $MoSe_2$ A exciton the heterojunction is 80% of that in monolayer $MoSe_2$. As a result, the true EF for the heterojunction in the region (I) should be ~0.5. With $\mu = 0$, we combine Equations (3), (7) and (8) to obtain the initial band alignment $\Delta E_{C0} = 49\ meV$, for the EF~ 0.5. This result is consistent with the calculation result of 60 meV[3].

With the quantitative analysis, we can also estimate at which voltage the type II to type I alignment transition will occur. We first formulate the relation between the gate voltage and chemical poetical:

$$V = \frac{n_1 + n_2}{C_{G1}} + \frac{n_2}{C_{G2}} + \frac{n_2}{C_{Q2}} \quad (10)$$

To get the transition point of the band alignment, we set $\Delta E_{C0} = 49\ meV$ and $\Delta E_C = 0$. By solving equation (10) and (4), the transition gate voltage can be calculated. It has been that the geometry capacitance C_{G1} can be as high as 4 μF based on a reported value of double of 0.885 nm[4], in the heavily doped regime. Even considering possible lack of optimization of the device structure, with an effective double layer as thick as ~ 8.85 nm, we would have an underestimated geometry capacitance $C_{G1} = 3.98 \times 10^{12} cm^2/eV$, which determines the transition voltage of ~1.4 V. As a result, at the heavily doped region as shown in the main text, corresponding to the gate voltage as high as ~ 4 V, should already be in the regime that the type II configuration has been switched to the type I configuration.

Supplementary Note 3. Derivation of the effective capacitance circuit model for the heterostructure device.

It has been shown by Luryi[5] that for a circuit shown in Supplementary Fig. 4a, the equivalent capacitance circuit is as shown in Supplementary Fig. 4b. In Supplementary Fig. 4a, the top and bottom plates are ideal metal plate, and the middle plate, Q, is a two-dimensional metal which does not screen the field completely. It could be 2D electron gas (2DEG) of a quantum well (QW), or in our case, a monolayer TMDC. As the quantum capacitance, $C_Q = g\frac{me^2}{\pi\hbar^2}$, is comparable to geometry capacitance C_1 and C_2, the energy to fill electron in the TMDC is not negligible and has to be taken into account. By minimizing the total energy of the system, including the energy to charge the two geometry capacitors (C_1 and C_2) and quantum capacitor (C_Q), it can be shown that the charge density in electrode 1 (σ_1) and electrode 2 (σ_2) follows the relation, $\sigma_2 = -\sigma_1 \frac{C_2}{C_2+C_Q}$, and the charge neutrality condition gives $\sigma_Q = -\sigma_1 - \sigma_2$. It is evident then that the equivalent circuit of Supplementary Fig. 4a should be Supplementary Fig. 4b.

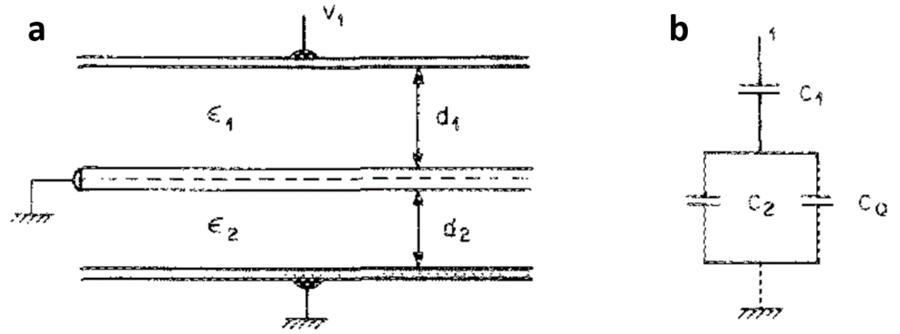

Supplementary Figure 4. Schematic of the capacitance model for a two-dimensional material sandwiched by two plates (a) and the equivalent circuit (b)[5].

Now, if we only have one monolayer TMDC device shown as schematically in Supplementary Fig. 5a, it will be equivalent to have the bottom plate (the grounded C_2) in Supplementary Fig. 5a to be placed at infinity, which gives a $C_2 = 0$, and the effective capacitance will be C_1 and C_Q in series. For the heterostructure device schematically shown in Supplementary Fig. 5c (also Fig. 1b of the main text), the bottom plate is not an ideal metal anymore and does not completely screen the electrical field. Instead, it is a quantum plate, just like the middle plate Q. In this case, we name the middle plate Q_1, and following our equivalent circuit of the monolayer device (Supplementary Fig. 5b), now the C_2 in Supplementary Fig. 5b should be replaced with geometry capacitance (C_2) and quantum capacitance (C_{Q2}) in series, and the resulting equivalent circuit will be as shown in Supplementary Fig. 5d (also the inset of Fig. 4 of the main text). Another way to understand is that, the extra voltage drop on the quantum capacitance 1 (C_{Q1}) has to be the same as the total voltage drop on the geometry capacitance (C_2) and quantum capacitance (C_{Q2}), as both the quantum plate 1 (the original middle plate Q in Supplementary Fig. 5a) and quantum plate 2 (the original bottom plate in Supplementary Fig. 5a) are both grounded, same as our experimental setup.

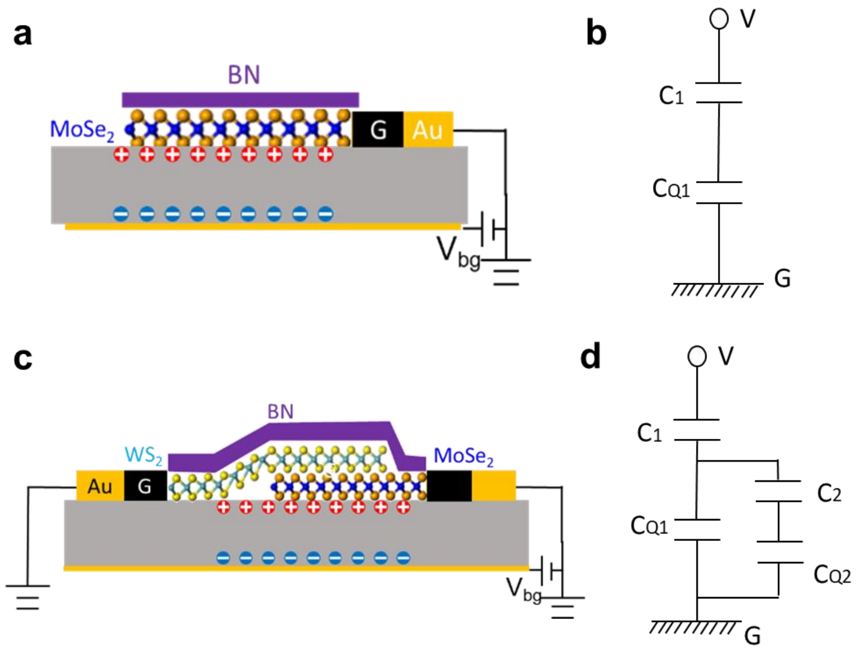

Suplementary Figure 5. (a) and (b) are the schematic for the monolayer $MoSe_2$ device and its corresponding equivalent capacitance circuit. (c) and (d) are the schematic for the heterostructure device and its corresponding equivalent capacitance circuit.

Supplementary Note 4. The low energy PL in the heterojunction originating from intralayer excitons in $MoSe_2$.

The fabrication of the hetero-bilayer of $MoSe_2$/WS_2 was through random stacking, which means that the K (K') valleys of the $MoSe_2$ and WS_2 are not aligned. This makes the charge transfer excitons (interlayer excitons) indirect bandgap in nature with momentum-mismatched electrons and holes. As a result, their radiative recombination rate and PL intensity should be strongly reduced. We have also performed systematic study to show that the low energy PL peak from the heterojunction is from the $MoSe_2$ A exciton.

We have performed the TRPL measurements at the $MoSe_2$ A exciton resonance for the monolayer $MoSe_2$ and $MoSe_2$/WS_2 heterojunction, as shown in Supplementary Fig. 6. It is evident that the PL lifetime from the $MoSe_2$ monolayer and the $MoSe_2$/WS_2 hetero-bilayer are almost the same (the one from the heterojunction is actually slightly shorter), contradicting the expected longer lifetime from the interlayer exciton due to spatial separation of electrons and holes. In addition, the fitting shows a fast decay component of ~ 5 ps (Supplementary Fig. 6), consistent with the previously reported lifetime of intralayer exciton but much shorter than the reported interlayer exciton lifetime in $MoSe_2$/WS_2 heterojunction (~ 80 ps) extracted from the transient absorption measurement[6].

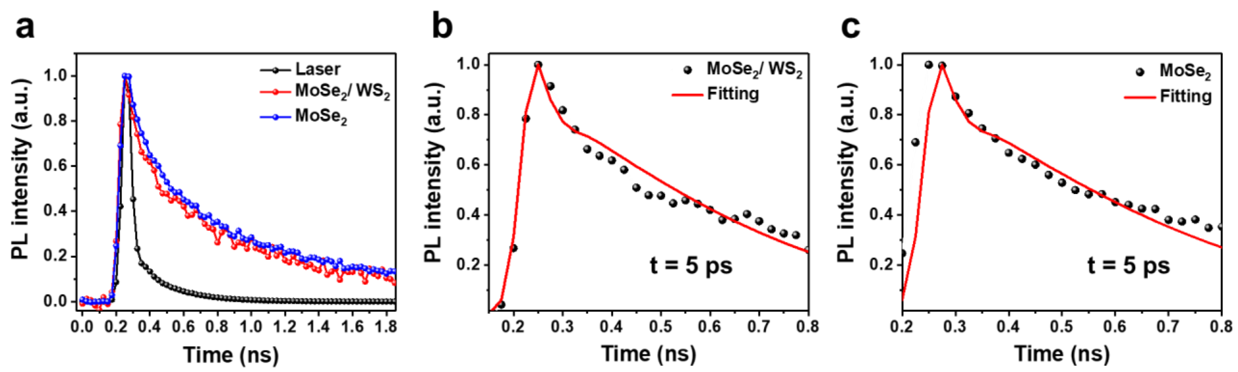

Supplementary Figure 6. The time-resolved PL of the monolayer $MoSe_2$ and $MoSe_2/WS_2$ junction at room temperature. (a) TRPL spectra from the heterojunction (red dots), monolayer $MoSe_2$ (blue dots), and response from the laser (black). (b) and (c) and convolution fittings to the experimental TRPL using the laser response as the kernel for the heterojunction and monolayer $MoSe_2$, respectively.

We have fabricated seven heterostructure devices on LaF_3 and three devices on SiO2/Si in total. The data from devices on the LaF_3 substrate are shown in Supplementary Fig. 7, and all the heterojunction showed PL quenching with no gate voltage applied, and the enhancement factor (EF) varying from 0.5 to 0.9. This systematic quenching behavior is different from the PL enhancement observed in previous work reporting the interlayer exciton in $MoSe_2/WS_2$ heterojunction[6], in which the PL enhancement was observed (EF>1) as the signature of the CT exciton (interlayer exciton).

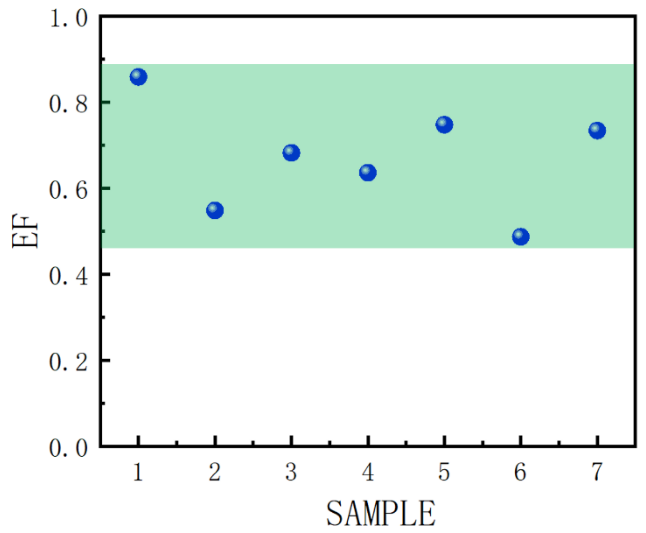

Supplementary Figure 7. PL EF for different $MoSe_2/WS_2$ heterojunctions on the LaF_3 substrate.

Due to the frozen ion effect in the LaF_3, we cannot tune the doping easily through gate voltage at low temperature and did not do extensive low-temperature study. However, we did fix the gate voltage at 0 V and measure PL from the heterojunction at room

temperature and 77 K under the same excitation condition. The results are shown in Supplementary Fig. 8. It is obvious that the PL at 77 K is much stronger than that at room temperature, opposite to the observation in the previous work of indirect bandgap interlayer exciton[7] and contradicts the expected behavior of indirect bandgap interlayer exciton.

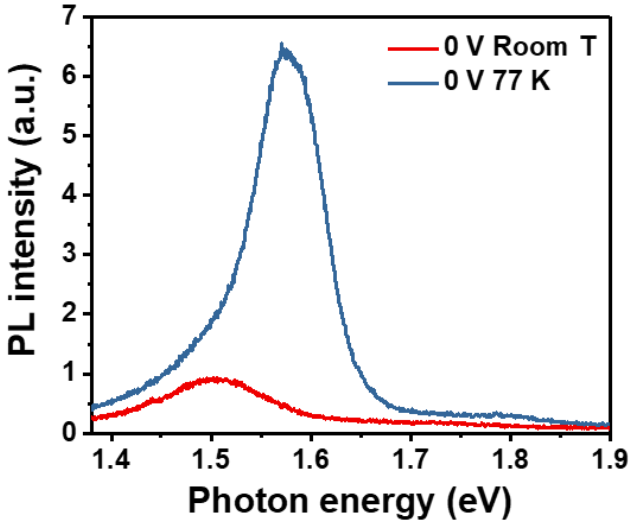

Supplementary Figure 8. PL spectra from the $MoSe_2/WSe_2$ junction on LaF_3 substrate at room temperature (red) and 77 K (blue).

Supplementary Note 5. Discussion of the trion effects in the doped region.

Charged exciton (trion) will emerge when WS_2 is sufficiently n doped. However, in the current model, the charged exciton (trion) plays the same role as the neutral exciton. For example, whether exciting an exciton or a trion in WS_2 would lead to the same amount of additional electron and hole density, and therefore the same amount of charge transfer between layers. As a result, the only relevant parameter here is the absorption of WS_2 at the excitation light energy, regardless of the nature of the excited state. The introduction of charged exciton does not change our theoretical understanding or our model.

Supplementary Note 6. Additional PLE spectra from another device on LaF_3.

Here we also provide a PLE study of a second device on LaF_3 in Supplementary Fig. 9, which shows similar results as Fig. 3 in the main text.

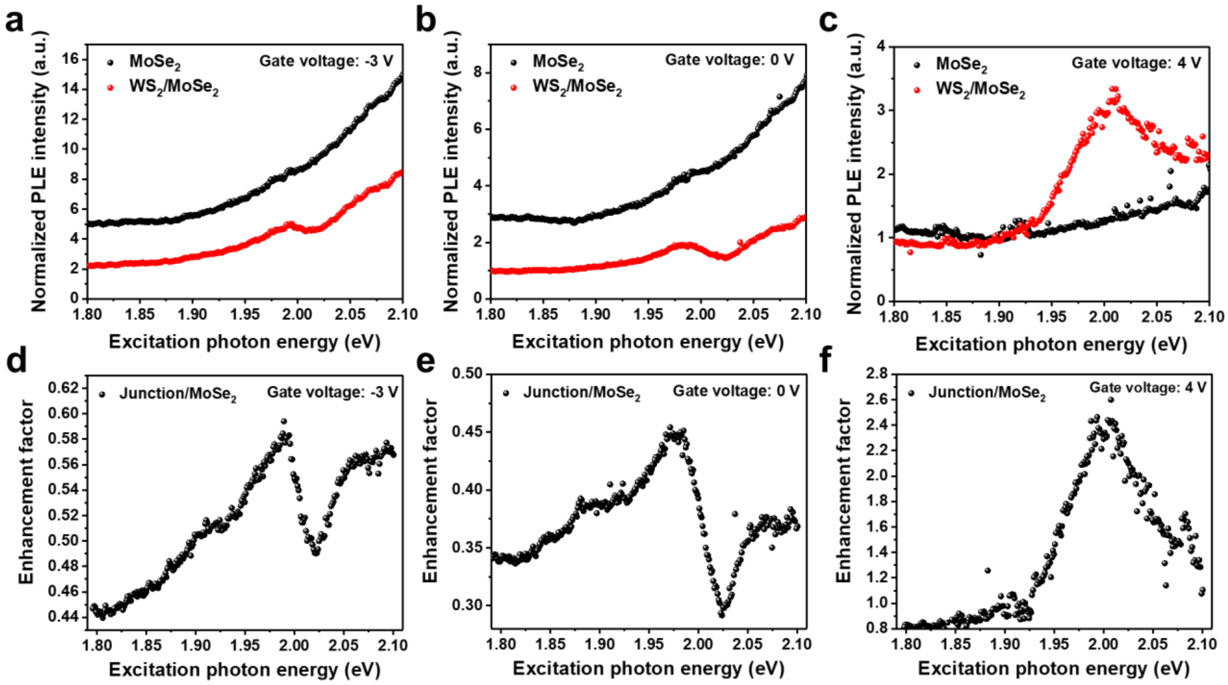

Supplementary Figure 9. PLE spectra and EF as a function of the excitation for a second device on LaF_3. (a)-(c) are the PL spectra for the gate voltage of -3 V, 0 V, and 4 V. The corresponding EF as a function of the excitation photon energy is shown in (d)-(f).

Supplementary Note 7. Spatially resolved PL mapping.

It is well-known that the stacked heterostructure devices would have spatial inhomogeneity. Therefore, we performed spatially resolved PL mapping for the heterojunction and monolayer $MoSe_2$ at different experimental conditions, shown in Supplementary Fig. 10. It is evident that at gate voltage -3 V (Supplementary Fig. 10a,b), when both $MoSe_2$ and WS_2 are intrinsic, the PL ($MoSe_2$ A exciton) from the heterojunction is also weaker than that from the monolayer $MoSe_2$, no matter whether the optical excitation can excite the WS_2 (2.33 eV) or not (1.80 eV). However, at the gate voltage of 4 V (Supplementary Fig. 10c,d), when both $MoSe_2$ and WS_2 are highly n-doped, the PL from the heterojunction is stronger than the monolayer $MoSe_2$ when the optical excitation excites WS_2 (2.33 eV). It is clear that there is spatial inhomogeneity, which will eventually give rise to the uncertainty in the enhancement factor. However, the overall trend of enhancement is evident.

It is worth noting that the PL from the heterojunction appears to be weaker than the monolayer $MoSe_2$ when the optical excitation is below the A exciton resonance of WS_2 (1.80 eV), while theoretically, it should be the same. The reason is that the quantum efficiency of the heterojunction is typically reduced, likely due to the different dielectric environment. The borderline of EF, ideally to be 1, usually varying from 0.8 to 0.9 in reality. There are also a few spots that PL remain relatively strong (red spots in Supplementary Fig. 10d), which we attribute to the poor coupling to the LaF_3 substrate,

and the gate voltage was not effectively applied to the sample at those spots.

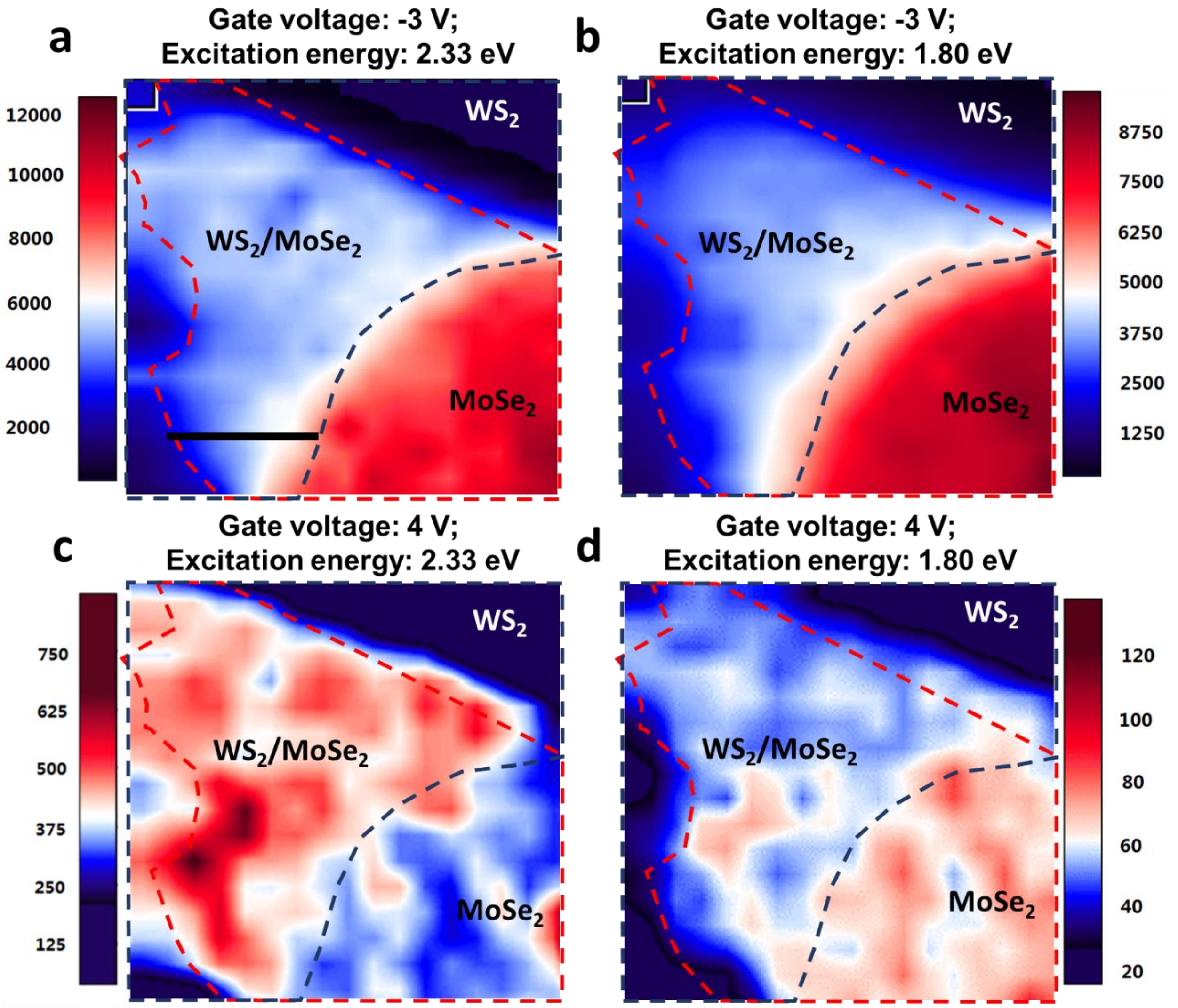

Supplementary Figure 10. Spatial PL mapping. (a) and (b) are the spatially resolved PL mapping at the gate voltage of -3 V with the photoexcitation centered at 2.33 eV and 1.80 eV, respectively. (c) and (d) are the spatially resolved PL mapping at the gate voltage of 4 V with the photoexcitation centered at 2.33 eV and 1.80 eV, respectively

Supplementary References

1. Li, Z. *et al.* Revealing the biexciton and trion-exciton complexes in BN encapsulated WSe_2. *Nat. Commun.* **9**, 3719 (2018).
2. Laturia, A., Van de Put, M. L. & Vandenberghe, W. G. Dielectric properties of hexagonal boron nitride and transition metal dichalcogenides: from monolayer to bulk. *npj 2D Mater. Appl.* **2**, 6 (2018).
3. Ruiz-Tijerina, D. A. & Fal'ko, V. I. Interlayer hybridization and moiré superlattice minibands for electrons and excitons in heterobilayers of transition-metal dichalcogenides. *Phys. Rev. B* **99**, 125424 (2019).
4. Wu, C. L. *et al.* Gate-Induced Metal-Insulator Transition in MoS_2 by Solid Superionic Conductor LaF_3. *Nano Lett.* **18**, 2387–2392 (2018).
5. Luryi, S. Quantum capacitance devices. *Appl. Phys. Lett.* **52**, 501–503 (1988).
6. Ceballos, F., Bellus, M. Z., Chiu, H.-Y. & Zhao, H. Probing charge transfer excitons in a $MoSe_2$–WS_2 van der Waals heterostructure. *Nanoscale* **7**, 17523–17528 (2015).
7. Kunstmann, J. *et al.* Momentum-space indirect interlayer excitons in transition-metal dichalcogenide van der Waals heterostructures. *Nat. Phys.* **14**, 801–805 (2018).
